# Aligning coding sequences with frameshift extension penalties

**DOI:** 10.1186/s13015-017-0101-4

**Published:** 2017-03-31

**Authors:** Safa Jammali, Esaie Kuitche, Ayoub Rachati, François Bélanger, Michelle Scott, Aïda Ouangraoua

**Affiliations:** 1 Département d’informatique, Faculté des Sciences, Université de Sherbrooke, Sherbrooke, QC J1K2R1 Canada; 2 Département de biochimie, Faculté de médecine et des sciences de la santé, Université de Sherbrooke, Sherbrooke, QC J1E4K8 Canada

**Keywords:** Coding DNA sequences pairwise alignment, Frameshifts, Dynamic programming

## Abstract

**Background:**

Frameshift translation is an important phenomenon that contributes to the appearance of novel coding DNA sequences (CDS) and functions in gene evolution, by allowing alternative amino acid translations of gene coding regions. Frameshift translations can be identified by aligning two CDS, from a same gene or from homologous genes, while accounting for their codon structure. Two main classes of algorithms have been proposed to solve the problem of aligning CDS, either by amino acid sequence alignment back-translation, or by simultaneously accounting for the nucleotide and amino acid levels. The former does not allow to account for frameshift translations and up to now, the latter exclusively accounts for frameshift translation initiation, not considering the length of the translation disruption caused by a frameshift.

**Results:**

We introduce a new scoring scheme with an algorithm for the pairwise alignment of CDS accounting for frameshift translation initiation and length, while simultaneously considering nucleotide and amino acid sequences. The main specificity of the scoring scheme is the introduction of a penalty cost accounting for frameshift extension length to compute an adequate similarity score for a CDS alignment. The second specificity of the model is that the search space of the problem solved is the set of all feasible alignments between two CDS. Previous approaches have considered restricted search space or additional constraints on the decomposition of an alignment into length-3 sub-alignments. The algorithm described in this paper has the same asymptotic time complexity as the classical Needleman–Wunsch algorithm.

**Conclusions:**

We compare the method to other CDS alignment methods based on an application to the comparison of pairs of CDS from homologous *human*, *mouse* and *cow* genes of ten mammalian gene families from the Ensembl-Compara database. The results show that our method is particularly robust to parameter changes as compared to existing methods. It also appears to be a good compromise, performing well both in the presence and absence of frameshift translations. An implementation of the method is available at https://github.com/UdeS-CoBIUS/FsePSA.

**Electronic supplementary material:**

The online version of this article (doi:10.1186/s13015-017-0101-4) contains supplementary material, which is available to authorized users.

## Background

Biological sequence alignment is a cornerstone of bioinformatics and is widely used in such fields as phylogenetic reconstruction, gene finding, genome assembly. The accuracy of the sequence alignments and similarity measures are directly related to the accuracy of subsequent analysis. CDS alignment methods have many important applications for gene tree and protein tree reconstruction. In fact, they are useful to cluster homologous CDS into groups of orthologous splicing isoforms [1, 2] and combine partial trees on orthology groups into a complete protein tree for a gene family [3, 4]. Aligning and measuring the similarity between homologous CDS requires to account for *frameshift (FS) translations* that cannot be detected at the amino acid (AA) level, but lead to a high similarity at the nucleotide level between functionnaly different sub-sequences.

FS translation consists in alternative AA translations of a coding region of DNA using different translation frames [5]. It is an important phenomenon resulting from different scenarios such as, insertion or deletion of a nucleotide sequence whose length is not a multiple of 3 in a CDS through alternative splicing [6, 7] or evolutionary genomic indels [8, 9], programmed ribosomal frameshifting [10], or sequencing errors [11]. Recent studies have reported the role of FS translations in the appearance of novel CDS and functions in gene evolution [6, 12]. FS translation has also been found to be linked to several diseases such as the Crohn’s disease [13]. The computational detection of FS translations requires the alignment of CDS while accounting for their codon structure. A classical approach for aligning two CDS used in most alignment tools [14, 15] consists in a three-step method, where the CDS are first translated into AA sequences using their actual coding frame, then AA sequences are aligned, and finally the AA alignment is back-translated to a CDS alignment. This approach does not account for alternative AA translations between two CDS and it leads to incorrect alignment of the coding regions subject to FS translation. The opposite problem of aligning protein sequences while recovering their hypothetical nucleotide CDS sequences and accounting for FS translation was also studied in several papers [16, 17].

Here, we consider the problem of aligning two CDS while accounting for FS translation, by simultaneously accounting for their nucleotide and AA sequences. The problem has recently regained attention due to the increasing evidence for alternative protein production through FS translation by eukaryotic gene families [18, 19].

The problem was first addressed by Hein et al. [20, 21] who proposed a DNA/protein model such that the score of an alignment between two CDS of length *n* and *m* is a combination of its score at the nucleotide level and its score at the AA level. They described a* O*(*n* ^2^ *m* ^2^) algorithm in [20], later improved to a *O*(*nm*) algorithm in [21] for computing an optimal score alignment, under the constraint that the search space of the problem is restricted. Arvestad [22] later proposed a CDS alignment scoring model with a *O*(*nm*) alignment algorithm accounting for codon structures and FS translations based on the concept of generalized substitutions introduced in [23]. In this model, the score of a CDS alignment depends on its decomposition into a concatenation of *codon fragment* alignments, such that a codon fragment of a CDS is defined as a substring of length *w*, $0\le w \le 5$. This decomposition into codon fragment alignments allows to define a score of the CDS alignment at the AA level. More recently, Ranwez et al. [18] proposed a simplification of the model of Arvestad limiting the maximum length of a codon fragment to 3. Under this model, a *O*(*nm*) CDS alignment algorithm was described and extended in the context of multiple sequence alignment [18]. In the models of Arvestad [22] and Ranwez et al. [18], several scores may be computed for the same alignment based on different decompositions into codon fragment alignments. The corresponding algorithms for aligning two CDS then consist in computing an optimal score decomposition of an alignment between the two CDS. This optimal score exclusively accounts for FS translation initiations, i.e a FS translation in an alignment is penalized by adding a constant FS cost, which only penalizes the initiation of the FS, not accounting for the length of this FS translation. However, taking account of FS translation lengths is important in order to increase the precision of CDS alignment scores, as these lengths induce more or less disruptions between the protein sequences.

In this paper, we propose the first alignment algorithm that accounts for both the initiation and the length of FS translations in order to compute the similarity scores of CDS alignments. The remaining of the paper is organized as follows. In the “Motivation”, we illustrate the importance of accounting for FS translation length when aligning CDS. In the “Preliminaries”, we give some preliminary definitions and we introduce a new CDS alignment scoring model with a self-contained definition of the score of an alignment penalizing both the initiation and the extension of FS translations. In the “Methods”, a dynamic programming algorithm for computing an optimal score alignment between two CDS is described. Finally, in the “Results”, we present and discuss the results of a comparison of our method with other CDS alignment methods for a pairwise comparison of CDS from homologous genes of ten mammalian gene families.

### Motivation: importance of accounting for FS translation length

The two main goals of aligning biological sequences are to evaluate the similarity and to identify similar regions between the sequences, used thereafter to realize molecular analyses such as evolutionary, functional and structural predictions. In practice, CDS alignment can be used to exhaustively identify the conserved features of a set of proteins. Thus, the definition of CDS similarity must account for sequence conservation and disruptions at both the nucleotide and the protein levels.

**Fig. 1:**
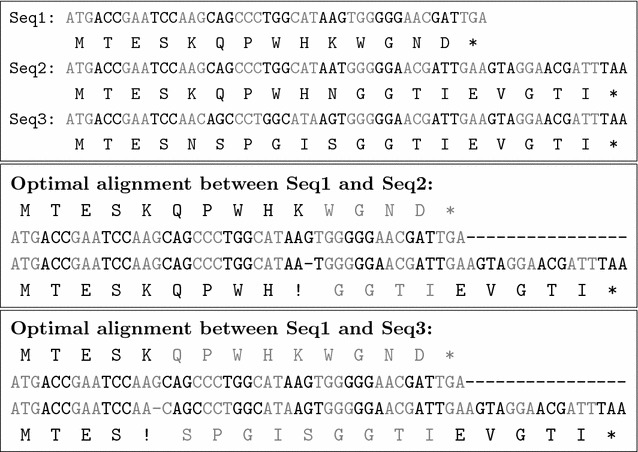
*Top* an example of three CDS Seq1, Seq2 and Seq3. *Middle* an optimal alignment between Seq1 and Seq2 with a FS translation region of length 15. *Bottom* an optimal alignment between Seq1 and Seq3 with a FS translation region of length 30

Figure 1 illustrates the importance of accounting for AA translations and FS translation length in order to compute an adequate similarity score for a CDS alignment. It describes an example of three CDS Seq1, Seq2 and Seq3. Seq1 has a length of 45. The CDS Seq2 has length 60 and is obtained from Seq1 by deleting the nucleotide ‘G’ at position 30 and adding 16 nucleotides at the end. The CDS Seq3 has length 60 and is obtained from Seq1 by deleting the nucleotide ‘G’ at position 15 and adding 16 nucleotides at the end.

When looking at the AA translations of Seq1, Seq2 and Seq3, we observe that the similarity between Seq2 and Seq1 is higher than the similarity between Seq3 and Seq1 at the protein level, because Seq1 and Seq2 share a longer AA prefix “M T E S K Q P W H” (amino acids in black characters in the alignments). However, the pairwise CDS alignment algorithms that do not account for the length of FS translations would return the same score for the two following optimal alignments of Seq1 with Seq2 and Seq1 with Seq3, penalizing only the initiation of one FS translation in both cases (positions marked with a “!” symbol in the alignments), and not penalizing the sequence disruptions at the protein level.

From an evolutionary point of view, a good scoring model for evaluating the similarity between two CDS in the presence of FS translations should then penalize not only the initiation of FS but also the length of FS translations extension (amino acids in gray characters in the alignments). The alignment of Seq1 with Seq2 would then have a higher similarity score than the alignment of Seq1 with Seq3.

### Preliminaries: score of CDS alignment

In this section, we formally describe a new definition of the score of a CDS alignment that penalizes both the initiation and the extension of FS translations.

#### **Definition 1**

[*Coding DNA sequence (CDS)*] A coding DNA sequence (CDS) is a DNA sequence on the alphabet of nucleotides $\Sigma _N=\{A,C,G,T\}$ whose length *n* is a multiple of 3. A coding sequence is composed of a concatenation of $\frac{n}{3}$ codons that are the words of length 3 in the sequence ending at positions 3*i*, $1 \le i \le \frac{n}{3}$. The AA translation of a CDS is a protein sequence of length $\frac{n}{3}$ on the alphabet $\Sigma _A$ of AA such that each codon of the CDS is translated into an AA symbol in the protein sequence.

Note that, in practice an entire CDS begins with a start codon “ATG” and ends with a stop codon “TAA”, “TAG” or “TGA”.

#### **Definition 2**

(*Alignment between DNA sequences*) An alignment between two DNA sequences *A* and *B* is a pair $(A',B')$ where $A'$ and $B'$ are two sequences of same length *L* derived by inserting gap symbols $'-'$ in *A* and *B*, such that $\forall i, ~1 \le i \le L, ~ A'[i] \ne ~'-'$ or $B'[i] \ne ~'-'$. Each position $i, ~1 \le i \le L$, in the alignment is called a column of the alignment.

Given an alignment $(A',B')$ of length *L* between two CDS *A* and *B*, let *S* be the sequence $A'$ or $B'$. We denote by $S[k~\ldots~l], ~1 \le k \le l \le L$, the substring of *S* going from position *k* to position *l*. $\left| {S[k \ldots l]} \right|$ denotes the number of letters in ${S[k \ldots l]}$ that are different from the gap symbol $'-'$. For example, if $A'=\texttt {ACCAT--GTAG}$ and $B'=\texttt {AC--TACGTAG}$, $|A'[4~..~8]| = |\texttt {AT--G}| = 3$. A codon of *A* or *B* is *grouped in the alignment* $(A',B')$ if its three nucleotides appear in three consecutive columns of the alignment. For example, the first codon ACC of *A* is grouped, while the first codon ACT of *B* is not grouped.

**Fig. 2:**
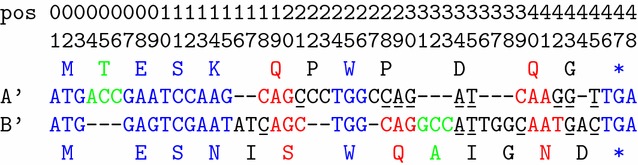
An alignment $(A',B')$ of length 48 between two CDS, *A* (13 codons) and *B* (14 codons). The* number arrays* indicate the positions of the consecutive alignment columns. Codons of *A* and *B* are* colored *according to the set to which they belong: IM codons in* blue color*, FSext codons in* red color*, InDel codons in* green color* and FSinit codons in* black color*. MFS nucleotides contained in FSinit codons are* underlined*

In the following, we give our definition of the score of an alignment $(A',B')$ between two CDS *A* and *B*. It is based on a partition of the codons of *A* (resp. *B*) into four sets depending on the alignment of codons (see Fig. 2 for an illustration):

1. The set of In-frame Matching codons (IM) contains the codons that are grouped in the alignment and aligned with a codon of the other CDS.
2. The set of Frameshift extension codons (FSext) contains the codons that are grouped in the alignment and aligned with a concatenation of three nucleotides that overlaps two codons of the other CDS.
3. The set of Deleted/Inserted codons (InDel) contains the codons that are grouped in the alignment and aligned with a concatenation of 3 gap symbols.
4. All other codons constitutes Frameshift initiation codons (FSinit). The set of Matching nucleotides in FSinit codons (MFS) contains all the nucleotides belonging to FSinit codons and aligned with a nucleotide of the other CDS.

The following notations and conventions are used in Definition 3 to denote the different sets of codons and nucleotides in *A* and *B*. The set of IM codons in *A* (resp. *B*) is denoted by $\texttt {IM}_{A\rightarrow B}$ (resp. $\texttt {IM}_{B\rightarrow A}$). The set of FSext codons in *A* (resp. *B*) is denoted by $\texttt {FSext}_{A\rightarrow B}$ (resp. $\texttt {FSext}_{B\rightarrow A}$). The set of InDel codons in *A* (resp. *B*) is denoted by $\texttt {InDel}_{A\rightarrow B}$ (resp. $\texttt {InDel}_{B\rightarrow A}$). The set of MFS nucleotides in *A* (resp. *B*) is denoted by $\texttt {MFS}_{A\rightarrow B}$ (resp. $\texttt {MFS}_{B\rightarrow A}$). In these sets, the codons of *A* and *B* are simply identified by the position (column) of their last nucleotide in the alignment. In this case, we always have $\texttt {IM}_{A\rightarrow B} = \texttt {IM}_{B\rightarrow A}$ as in the example below. The MFS nucleotides are also identified by their positions in the alignment.

For example, for the alignment depicted in Fig. 2, the composition of the different sets are: $\texttt {IM}_{A\rightarrow B} = \texttt {IM}_{B\rightarrow A} = \{ 3, 9, 12, 15, 26, 48\}$; $\texttt {FSext}_{A\rightarrow B} = \{20, 41\}$; $\texttt {InDel}_{A\rightarrow B} = \{ 6 \}$; $\texttt {MFS}_{A\rightarrow B} = \{21,28,29,30,34,35,42,43,45\}$; $\texttt {FSext}_{B\rightarrow A} = \{ 21, 30, 42\}$; $\texttt {InDel}_{B\rightarrow A} = \{ 33\}$; and $\texttt {MFS}_{B\rightarrow A} = \{ 18, 34, 35, 39, 43, 45\}.$

In the alignment scoring model described in Definition 3, the substitutions of IM and FSext codons are scored using an AA scoring function $s_{aa}$ such that aligned codons with silent nucleotide mutations get the same score as identity. A fixed FS extension cost denoted by fs_extend_cost is added for each FSext codon. The insertions/deletions of InDel codons are scored by adding a fixed gap cost denoted by gap_cost for each InDel codon. The alignment of MFS nucleotides are scored independently from each other, using a nucleotide scoring function $s_{an}$. The insertions or deletions of nucleotides in FSinit codons are responsible for the initiation of FS translations. They are then scored by adding a fixed FS opening cost denoted by fs_open_cost for each FSinit codon. Note that, by convention, the values of all penalty costs for gap and FS (gap_cost, fs_open_cost, fs_extend_cost) are negative. Note also that the scoring scheme assumes that the AA and the nucleotide scoring functions, $s_{aa}$ and $s_{an}$, are symmetric.

#### **Definition 3**

(*Score of an alignment*) Let $(A',B')$ be an alignment of length *L* between two CDS *A* and *B*. The score of the alignment $(A',B')$ is defined by:

$$\begin{aligned} \begin{array}{lll} \texttt {score}(A',B') &{} =&{} \sum_{k \in \texttt {IM}_{A\rightarrow B}}{s_{aa}(A'[k-2~\ldots~k],B'[k-2~\ldots~k])} ~ +\\ &{} &{} \sum _{k \in \texttt {FSext}_{A\rightarrow B}}{( \frac{s_{aa}(A'[k-2~\ldots~k],B'[k-2~\ldots~k])}{2} + \texttt {fs\_extend\_cost} )} ~ +\\ &{} &{} |\texttt {InDel}_{A\rightarrow B}| * \texttt {gap\_cost} ~ +\\ &{} &{} (\frac{|A|}{3} - |\texttt {IM}_{A\rightarrow B}| - |\texttt {FSext}_{A\rightarrow B}| - |\texttt {InDel}_{A\rightarrow B}|) * \texttt {fs\_open\_cost} ~ + \\ &{} &{} \sum _{k \in \texttt {MFS}_{A\rightarrow B}}{\frac{s_{an}(A'[k],B'[k])}{2}} ~ + \\ &{} &{} \sum _{k \in \texttt {FSext}_{B\rightarrow A}}{ (\frac{s_{aa}(B'[k-2~\ldots~k],A'[k-2~\ldots~k])}{2} + \texttt {fs\_extend\_cost})} ~+\\ &{} &{} |\texttt {InDel}_{B\rightarrow A}| * \texttt {gap\_cost} ~+\\ &{} &{} (\frac{|B|}{3} - |\texttt {IM}_{B\rightarrow A}| - |\texttt {FSext}_{B\rightarrow A}| - |\texttt {InDel}_{B\rightarrow A}|) * \texttt {fs\_open\_cost} ~+ \\ &{} &{} \sum _{k \in \texttt {MFS}_{B\rightarrow A}}{\frac{s_{an}(B'[k],A'[k])}{2}}\\ \end{array} \end{aligned}$$

## Methods

In this section, we describe a *O*(*nm*) time and space complexity algorithm that solves the problem of finding a maximum score alignment between two CDS *A* and *B* of lengths *n* and *m*. Similarly to other classical sequence alignment algorithms [24], we use dynamic programming tables that are indexed by the pairs of prefixes of the two CDS. The table *D* stores the maximum scores of the alignments between prefixes of *A* and *B*. The table $D_F$ is used to account for potential cases of FS extensions that are counted subsequently.

### **Definition 4**

(*Dynamic programming tables*) Given two CDS *A* and *B* as input, the algorithm uses two dynamic programming tables *D* and $D_F$ of size $(n+1)\times (m+1)$. The cell *D*(*i*, *j*) contains the maximum score of an alignment between the prefixes $A[1\ldots i]$ and $B[1\ldots j]$. The table $D_F$ is filled only for values of *i* and *j* such that $i (mod~3) = 0$ or $j (mod~3) = 0$. If $i (mod~3) \ne 0$ (resp. $j (mod~3) \ne 0$), the cell $D_F(i,j)$ contains the score of an alignment between the prefixes $A[1~\ldots~i+\alpha ]$ and $B[1~\ldots~j+\alpha ]$ where $\alpha = (3-i) (mod~3)$ (resp. $\alpha = (3-j) (mod~3)$). The table $D_F$ is filled as follows:

- If $$i (mod~3) = 0$$ and $$j (mod~3) = 0$$, $$D_F(i,j) = D(i,j)$$.
- If $i (mod~3) = 0$ and $j (mod~3) = 2$, or $i (mod~3) = 2$ and $j (mod~3) = 0$, $D_F(i,j)$ contains the maximum score of an alignment between $A[1~\ldots~i+1]$ and $B[1~\ldots~j+1]$ such that $A[i+1]$ and $B[j+1]$ are aligned together and half of the score for aligning $A[i+1]$ with $B[j+1]$ is subtracted.
- If $i (mod~3) = 0$ and $j (mod~3) = 1$, or $i (mod~3) = 1$ and $j (mod~3) = 0$, $D_F(i,j)$ contains the maximum score of an alignment between $A[1~\ldots~i+2]$ and $B[1~\ldots~j+2]$ such that $A[i+1]$,$B[j+1]$ and $A[i+2]$,$B[j+2]$ are aligned together and half of the scores of aligning $A[i+2]$ with $B[j+2]$ and $A[i+1]$ with $B[j+1]$ is subtracted.

### **Lemma 1**

(Filling up table D)

1. *If* $$i (mod~3) = 0$$ *and* $$j (mod~3) = 0$$
  $$\begin{aligned} D(i,j) = \max \left\{ \begin{array}{ll} 1. &{} s_{aa}(A[i-2~\ldots~i],B[j-2~\ldots~j]) + D(i-3,j-3)\\ 2. &{} s_{an}(A[i],B[j]) + s_{an}(A[i-1],B[j-1]) + D(i-3,j-2) + 2 * \texttt {fs\_open\_cost}\\ 3. &{} s_{an}(A[i],B[j]) + s_{an}(A[i-2],B[j-1]) + D(i-3,j-2) + 2 * \texttt {fs\_open\_cost}\\ 4. &{} s_{an}(A[i],B[j]) + D(i-3,j-1) + 2* \texttt {fs\_open\_cost}\\ 5. &{} s_{an}(A[i],B[j]) + s_{an}(A[i-1],B[j-1]) + D(i-2,j-3) + 2 * \texttt {fs\_open\_cost}\\ 6. &{} s_{an}(A[i],B[j]) + s_{an}(A[i-1],B[j-2]) + D(i-2,j-3) + 2 * \texttt {fs\_open\_cost}\\ 7. &{} s_{an}(A[i],B[j]) + D(i-1,j-3) + 2* \texttt {fs\_open\_cost}\\ 8. &{} s_{an}(A[i],B[j]) + D(i-1,j-1) + 2 * \texttt {fs\_open\_cost}\\ 9. &{} \frac{s_{an}(A[i-1],B[j])}{2} + \frac{s_{an}(A[i-2],B[j-1])}{2} + D_F(i-3,j-2) + \texttt {fs\_open\_cost}\\ 10. &{} s_{an}(A[i-1],B[j]) + D(i-3,j-1) + 2 * \texttt {fs\_open\_cost}\\ 11. &{} \frac{s_{an}(A[i-2],B[j])}{2} + D_F(i-3,j-1) + \texttt {fs\_open\_cost}\\ 12. &{} \texttt {gap\_cost} + D(i-3,j) \\ 13. &{} D(i-1,j) + \texttt {fs\_open\_cost}\\ 14. &{} \frac{s_{an}(A[i],B[j-1])}{2} + \frac{s_{an}(A[i-1],B[j-2])}{2} + D_F(i-2,j-3) + \texttt {fs\_open\_cost}\\ 15. &{} s_{an}(A[i],B[j-1]) + D(i-1,j-3) + 2 * \texttt {fs\_open\_cost}\\ 16. &{} \frac{s_{an}(A[i],B[j-2])}{2} + D_F(i-1,j-3) + \texttt {fs\_open\_cost}\\ 17. &{} \texttt {gap\_cost} + D(i,j-3) \\ 18. &{} D(i,j-1) + \texttt {fs\_open\_cost}\\ \end{array} \right. \end{aligned}$$
2. *If* $$i (mod~3) = 0$$ *and* $$j (mod~3) \ne 0$$
  $$\begin{aligned} D(i,j) = \max \left\{ \begin{array}{ll} 1. &{} \frac{s_{aa}(A[i-2~\ldots~i],B[j-2~\ldots~j])}{2} + D_F(i-3,j-3) + \texttt {fs\_extend\_cost}\\ &{} + \frac{s_{an}(A[i],B[j])}{2} (+ \frac{s_{an}(A[i-1],B[j-1])}{2} ~if ~j-1 (mod~3) \ne 0)\\ 2. &{} s_{an}(A[i],B[j]) + s_{an}(A[i-1],B[j-1]) + D(i-3,j-2) + \texttt {fs\_open\_cost} \\ &{} (+ \texttt {fs\_open\_cost} ~if ~j-1 (mod~3) = 0)\\ 3. &{} s_{an}(A[i],B[j]) + s_{an}(A[i-2],B[j-1]) + D_F(i-3,j-2) + \texttt {fs\_open\_cost}\\ &{} (- \frac{s_{an}(A[i-2],B[j-1])}{2} ~if ~j-1 (mod~3) = 0)\\ 4. &{} s_{an}(A[i],B[j]) + D(i-3,j-1) + \texttt {fs\_open\_cost}\\ 5. &{} s_{an}(A[i],B[j]) + D(i-1,j-1) + \texttt {fs\_open\_cost}\\ 6. &{} s_{an}(A[i-1],B[j]) + s_{an}(A[i-2],B[j-1]) + D_F(i-3,j-2) + \texttt {fs\_open\_cost}\\ &{} (- \frac{s_{an}(A[i-2],B[j-1])}{2} ~if ~j-1 (mod~3) = 0)\\ 7. &{} s_{an}(A[i-1],B[j]) + D(i-3,j-1) + \texttt {fs\_open\_cost}\\ 8. &{} s_{an}(A[i-2],B[j]) + D(i-3,j-1) + \texttt {fs\_open\_cost}\\ 9. &{} \texttt {gap\_cost} + D(i-3,j) \\ 10. &{} D(i-1,j) + \texttt {fs\_open\_cost}\\ 11. &{} D(i,j-1)\\ \end{array} \right. \end{aligned}$$
3. *If* $i (mod~3) \ne 0$ *and* $j (mod~3) = 0$,* the equation is symmetric to the previous case*.
4. *If* $$i (mod~3) \ne 0$$ *and* $$j (mod~3) \ne 0$$ $$D(i,j) = \max \left\{ \begin{array}{ll} 1. &{} s_{an}(A[i],B[j]) + D(i-1,j-1)\\ 2. &{} D(i-1,j)\\ 3. &{} D(i,j-1)\\ \end{array} \right.$$

**Fig. 3:**
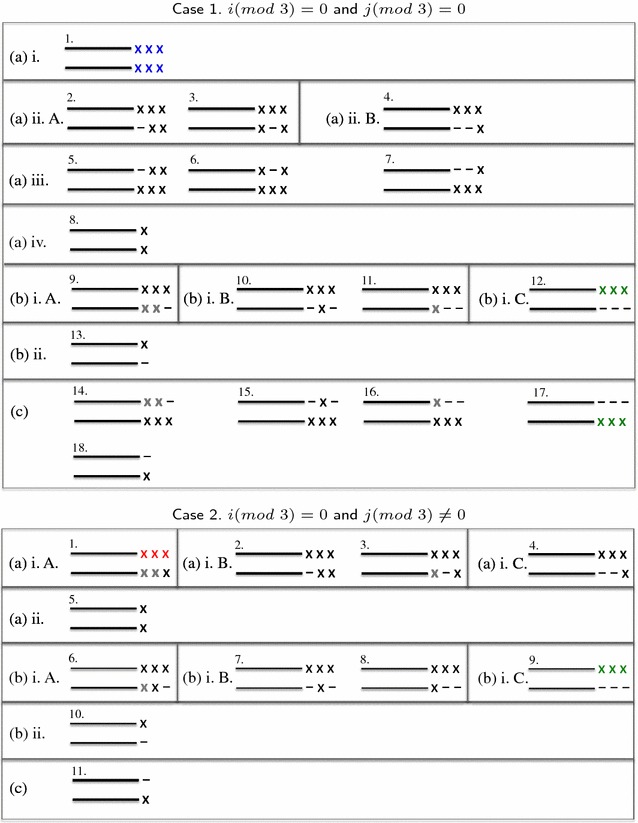
Illustration of the configurations of alignment considered in Lemma 1 for computing *D*(*i*, *j*) in cases 1 and 2. The* right-most* nucleotides of the sequences $A[1\ldots i]$  and $B[1\ldots j]$ *A*[1 .. *i*] and *B*[1 .. *j*] are represented using the character x. The nucleotides are* colored* according to the type of the codon to which they belong : IM codons in* blue color*, FSext codons in *red color*, InDel codons in* green color* and FSinit codons in* black color*. The nucleotides that appear in* gray color* are those belonging to codons whose type has not yet been decided. In such case, the table $D_F$ is used in order to decide of the type of these codons subsequently and adjust the score accordingly

The proof of Lemma 1 is given in the Additional file 1. Figure 3 illustrates the configurations of alignment considered in Lemma 1 for computing *D*(*i*, *j*) for cases 1 and 2.

### **Lemma 2**

(Filling up table $D_F$)

1. *If* $$i (mod~3) = 0$$ *and* $$j (mod~3) = 0$$
$$D_F(i,j) = D(i,j)$$
2. *If* $$i (mod~3) = 2$$ *and* $$j (mod~3) = 0$$
  $$\begin{aligned} D_F(i,j) = \max \left\{ \begin{array}{ll} 1. &{} \frac{s_{aa}(A[i-1~\ldots~i+1],B[j-1~\ldots~j+1])}{2} + D_F(i-2,j-2) + \texttt {fs\_extend\_cost}\\ 2. &{} \frac{s_{an}(A[i+1],B[j+1])}{2} + s_{an}(A[i],B[j]) + D(i-2,j-1) + 2 * \texttt {fs\_open\_cost}\\ 3. &{} \frac{s_{an}(A[i+1],B[j+1])}{2} + \frac{s_{an}(A[i-1],B[j])}{2} + D_F(i-2,j-1) + \texttt {fs\_open\_cost}\\ 4. &{} \frac{s_{an}(A[i+1],B[j+1])}{2} + D(i-2,j) + \texttt {fs\_open\_cost}\\ 5. &{} \frac{s_{an}(A[i+1],B[j+1])}{2} + D(i,j) + \texttt {fs\_open\_cost}\\ \end{array} \right. \end{aligned}$$
3. *If* $i (mod~3) = 0$ *and* $j (mod~3) = 2$,* the equation is symmetric to the previous case*.
4. *If* $$i (mod~3) = 1$$ *and* $$j (mod~3) = 0$$
  $$\begin{aligned} D_F(i,j) = \max \left\{ \begin{array}{ll} 1. &{} \frac{s_{aa}(A[i~\ldots~i+2],B[j~\ldots~j+2])}{2} + D_F(i-1,j-1) + \texttt {fs\_extend\_cost}\\ 2. &{} \frac{s_{an}(A[i+2],B[j+2])}{2} + \frac{s_{an}(A[i+1],B[j+1])}{2} + D(i-1,j) + \texttt {fs\_open\_cost}\\ 3. &{} \frac{s_{an}(A[i+2],B[j+2])}{2} + \frac{s_{an}(A[i+1],B[j+1])}{2} + D(i,j) + \texttt {fs\_open\_cost}\\ \end{array} \right. \end{aligned}$$
5. *If* $i (mod~3) = 0$ *and* $j (mod~3) = 1$,* the equation is symmetric to the previous case*.

### *Proof of Lemma 2*

The proof follows from Lemma 1.

1. If $i (mod~3) = 0$ and $j (mod~3) = 0$, this case is trivial.
2. If $i (mod~3) = 2$  and $j (mod~3) = 0$, then $i+1 (mod~3) = 0$ and $j+1 (mod~3) = 1 \ne 0$. The five cases follow from the application of Lemma 1, case 2 for computing $D(i+1,j+1)$, and by keeping only the cases where $A[i+1]$ and $B[j+1]$ are aligned together (cases 1, 2, 3, 4, 5 among the 11 cases). However, in each of the cases, we must subtract half of the score of aligning $B[i+1]$ with $A[j+1]$ $\left(\frac{s_{an}(A[i+1],B[j+1])}{2}\right)$, because this score will be added subsequently.
3. If $i (mod~3) = 0$ and $j (mod~3) = 2$, the proof is symmetric to the previous case.
4. If $i (mod~3) = 1$ and $j (mod~3) = 0$, then $i+2 (mod~3) = 0$ and $j+2 (mod~3) = 2 \ne 0$. Here again, the three cases follow from the application of Lemma 1, case 2 for computing $D(i+2,j+2)$ and by keeping only the cases where $A[i+1]$, $B[i+1]$ and $A[i+2]$, $B[i+2]$ can be aligned together (cases 1, 2, 5 among the 11 cases). However, in each of the cases, we must subtract half of the scores of aligning $B[i+2]$ with $A[j+2]$ and aligning $B[i+1]$ with $A[j+1]$ $\left(\frac{s_{an}(A[i+2],B[j+2])}{2}, \frac{s_{an}(A[i+1],B[j+1])}{2}\right)$, because theses scores will be added subsequently.
5. If $i (mod~3) = 0$ and $j (mod~3) = 1$, the proof is symmetric to the previous case.

$$\square$$

The alignment algorithm using Lemmas 1 and 2 is described in the next theorem.

### **Theorem 1**

(Computing a maximum score alignment)* Given two CDS* *A* *and* *B* *of lengths* *n* *and* *m*,* a maximum score alignment between* *A* *and* *B* *can be computed in time and space* *O*(*nm*),* using the following algorithm*.

$$\begin{aligned}&\texttt {Algorithm \quad Align(A,B)}\\&\texttt {~~for \quad i = 0 \quad to \quad n \quad do}\\&\qquad \qquad D(i,0) = floor\left(\frac{i}{3}\right) * \texttt {gap\_cost}\\&\qquad \qquad D_F(i,0) = D(i,0) + \left\{ \begin{array}{ll} \frac{s_{an}(A[i+1],B[1])}{2} + \frac{s_{an}(A[i+2],B[2])}{2} + \texttt {fs\_open\_cost}, & \quad\texttt {if ~i (mod~3) = 1}\\ \frac{s_{an}(A[i+1],B[1])}{2} + \texttt {fs\_open\_cost}, & \quad\texttt {if ~i (mod~3) = 2} \end{array} \right. \end{aligned}$$

$$\begin{aligned}&\texttt {for \quad j = 0 \quad to \quad m \quad do}\\&\qquad \qquad D(0,j) = floor\left(\frac{j}{3}\right) * \texttt {gap\_cost}\\&\qquad \qquad D_F(0,j) = D(0,j) + \left\{ \begin{array}{ll} \frac{s_{an}(A[1],B[j+1])}{2} + \frac{s_{an}(A[2],B[j+2])}{2} + \texttt {fs\_open\_cost}, &{}\quad \texttt {if ~j (mod~3) = 1}\\ \frac{s_{an}(A[1],B[j+1])}{2} + \texttt {fs\_open\_cost}, & \quad\texttt {if ~j (mod~3) = 2}\\ \end{array} \right. \\ & \texttt {for \quad i = 0 \quad to \quad n \quad do}\\&\texttt { \quad for \quad j = 0 \quad to \quad m \quad do}\\&\qquad \qquad \texttt {compute \quad D(i,j) \quad using \quad Lemma 1}\\&\qquad \qquad \texttt {compute} \quad \,D_{F} (i,j)\, \texttt {\quad using \quad Lemma 2, ~if ~i (mod~3) = 0 ~or ~j (mod~3) = 0} \end{aligned}$$

### *Proof of Theorem 1*

The proof relies on two points: (1) the algorithm computes the maximum score of an alignment between *A* and *B* and (2) the algorithm runs with an *O*(*nm*) time and space complexity.

1. The validity of the algorithm, i.e. the fact that it fills the cells of the tables *D* and $D_F$ according to Definition 4, follows from five points.
  The initialization of the tables is a direct consequence of Definition 4.
  Lemmas 1 and 2.
  The couples (*i*, *j*) of prefixes of *A* and *B* that need to be considered in the algorithm are all the possible couples for *D*(*i*, *j*) and only the couples such that $i (mod~3) = 0$ or $j (mod~3) = 0$ for $D_F(i,j)$ [see all the cases in which the table $D_F$ is used in Lemmas 1 (7 cases) and 2 (3 cases)].
  The couples (*i*, *j*) of prefixes of *A* and *B* are considered in increasing order of length and *D*[*i*, *j*] is computed before $D_F[i,j]$ in the cases where $i (mod~3) = 0$ or $j (mod~3) = 0$.
  A backtracking of the algorithm allows to find a maximum score alignment between *A* and *B*.
2. The time and space complexity of the algorithm is a direct consequence of the number of cells of the tables *D* and $D_F$, $2 \times (n+1) \times (m+1)$. Each cell is filled in constant time. The exact formula for the computational complexity of the algorithm is computed below.

**Table:** 

	18	$\times$	$\frac{nm}{9}$	For $\frac{nm}{9}$ calls of the case 1 of Lemma 1
+	11	$\times$	$2\times \frac{nm}{3}$	For $2\times \frac{nm}{3}$ calls of the cases 2 or 3 of Lemma 1
+	3	$\times$	$\frac{4nm}{9}$	For $\frac{4nm}{9}$ calls of the case 4 of Lemma 1
+	1	$\times$	$\frac{nm}{9}$	For $\frac{nm}{9}$ calls of the case 1 of Lemma 2
+	5	$\times$	$2 \times \frac{nm}{9}$	For $2\times \frac{nm}{9}$ calls of the cases 2 and 3 of Lemma 2
+	3	$\times$	$2 \times \frac{nm}{9}$	For $2\times \frac{nm}{9}$ calls of the cases 4 and 5 of Lemma 2
Total = 12.55 nm

$$\square $$

## Results and discussion

We implemented the present CDS alignment algorithm with an affine gap penalty scheme [25] such that the penalty for a concatenation of *k* inserted (resp. deleted) codons is $\texttt {gap\_open\_cost} + k * \texttt {gap\_cost}$, such that $\texttt {gap\_open\_cost}$ is a negative penalty cost for gap initiations. This was done by adding two dynamic programming tables $G_A$ and $G_B$ such that the cell $G_A(i,j)$ (resp. $G_B(i,j)$) contains the maximum score of an alignment between the prefixes $A[1\ldots i]$ $B[1\ldots j]$where the codon $A[i-2~\ldots~i]$ (resp. $B[j-2~\ldots~i]$) is an InDel codon.

### Data

**Fig. 4:**
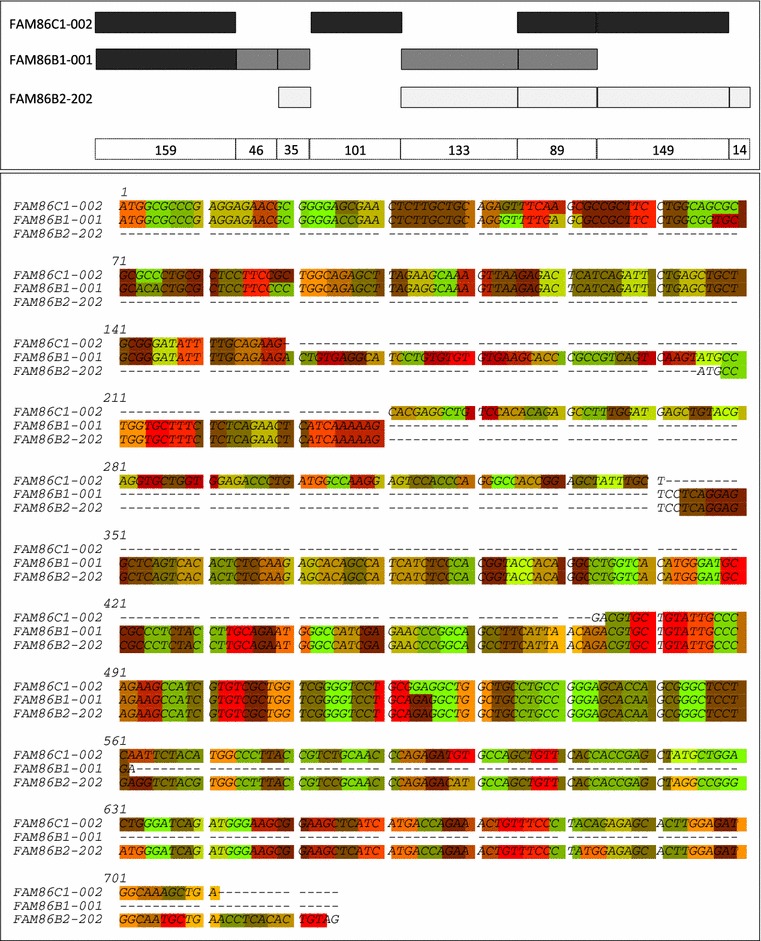
*Top rough* representation of the real alignment of CDS *FAM86C1-002*, *FAM86B1-001* and *FAM86B2-202*.* Rectangular colored portions* represent concatenations of nucleotides in the alignment while* blank portions* represent concatenations of gap symbols. The lengths of the alignment portions are given at the* bottom*. The* colors* of the nucleotide regions indicate the coding frame in which they are translated, taking the frame of CDS *FAM86C1-002* as reference. For example, there is a nucleotide region of length 89 shared by the three CDS and translated in 3 different coding frames. *Bottom* real alignment of three CDS (figure obtained using the visualization software seaview [29]). Nucleotides are* colored* according to the codon structure of the first CDS *FAM86C1-002*

We evaluated the algorithm through applications on a mammalian dataset containing CDS sequences from ten gene families obtained from the database Ensembl-Compara version 83 [26]. The first gene family named “FAM86” is such that three CDS from three of its paralogous human genes were shown in [6] to share a common FS region translated in three different frames in the three CDS (see Fig. 4 for an illustration of the multiple alignment of these three CDS). The nine other families are the nine smallest (in term of the overall length of CDS) of fifteen gene families listed in [12] where they were shown to display one FS translation region between some pairs of CDS. For each gene family, the CDS of all *human*, *mouse* and *cow* genes belonging to the family and satisfying Definition 1 were downloaded. The overall number of distinct pairs of CDS within the ten gene families is 4011. Table 1 gives the details about the content and size of the ten gene families (The CDS of the ten gene families are provided in the Additional file 2).

**Table 1:** Detailed description of the ten gene families of the mammalian dataset

Gene family	Human gene	# of genes	# of CDS	Length	$\frac{N*(N-1)}{2}$
I (FAM86)	ENSG00000118894	6	14	10335	91
II (HBG017385)	ENSG00000143867	6	10	8988	45
III (HBG020791)	ENSG00000179526	6	10	11070	45
IV (HBG004532)	ENSG00000173020	17	33	52356	528
V (HBG016641)	ENSG00000147041	13	33	64950	528
VI (HBG014779)	ENSG00000233803	28	44	45813	946
VII (HBG012748)	ENSG00000134545	24	44	28050	946
VIII (HBG015928)	ENSG00000178287	5	19	5496	171
IX (HBG004374)	ENSG00000140519	13	30	36405	435
X (HBG000122)	ENSG00000105717	11	24	27081	276
Total number of pairs of CDS					4011

For each gene family, the family identifier used in [6] or [12], the Ensembl identifier of a *human* gene member of the family, the number of *human*, *mouse* and *cow* genes in the family, the total number of CDS of these genes, the total sum of lengths of these CDS and the number of distinct pairs of CDS are given

### Evaluation strategies

We compared the accuracy of five pairwise global alignment methods, including the present method, for computing CDS alignments in the presence or absence of FS translation between the compared CDS. The five methods vary according to the alignment algorithm used, either the present CDS alignment algorithm called FsePSA allowing to penalize both FS translation initiation and extension, or the CDS alignment algorithm called MACSE [18] penalizing FS translation initiation, or the Needleman–Wunsch (NW) sequence alignment algorithm [24] penalizing neither. Table 2 summarizes the alignment algorithm and the values of parameters used for each of the five methods.

**Table 2:** Description of the five methods considered in the experiment

Method	Alignment approach and specific parameters	FS initiation cost	Other parameters
fse	Present approach $\texttt {fs\_extend\_cost} =$ $\,-1; -0.5; -0.2$	$\texttt {fs\_open\_cost} =$ −10; −20; −30	$\texttt {AA \quad gap\_open\_cost} = -11$ $\texttt {AA \quad gap\_cost} = -1$ $s_{aa} =\,\texttt {BLOSUM62 \quad matrix}$ $s_{an} =\,$ +1/-1 match/mismatch
fse0	Present approach $\texttt {fs\_extend\_cost} = 0$		
macse_p	Ranwez et al. [18] $\texttt {stop\_cost} = -100$		
needleprot	NW [24] at AA level	Not applicable	
needlenuc	NW [24] at NT level	Not applicable	$\texttt {NT \quad gap\_open\_cost} = -5$
			$$\texttt {NT \quad gap\_cost} = -2$$
			$s_{an} =$ +2/-3
			match/mismatch

For each method, the alignment approach and the values of specific and common parameters are given

The present CDS alignment algorithm is used in two of the five methods, namely fse and fse0. These two methods differ according to the value given to the parameter $\texttt {fs\_extend\_cost}$, either $\texttt {fs\_extend\_cost < 0}$ ($-1$, $-0.5$ or $-0.2$) for the method fse penalizing FS translation extension, or $\texttt {fs\_extend\_cost} = 0$ for the method fse0 not penalizing FS translation extension. The pairwise version of MACSE [18] is used in the method called macse_p. The NW alignment algorithm is used in the last two methods, the method called needlenuc computing scores and alignments at the nucleotide level and the method called needleprot at the AA level. For all methods using both the amino acid and nucleotide scoring functions $s_{aa}$ and $s_{an}$, $s_{an}$ was fixed to +1/-1 for match/mismatch, so that the overall score of 3 consecutive nucleotide identities in an alignment scores less than the smallest identity score in $s_{aa}$. All other parameters shared by several methods were given the same value for all methods. In particular, for the three methods fse, fse0 and macse_p penalizing FS translation initiation, the parameter $\texttt {fs\_open\_cost}$ was given the values $-10$, $-20$ or $-30$. All other parameters were fixed to the default values for the NW algorithm implementation of NCBI Blast at the nucleotide and AA levels [27].

We used the five methods to compute pairwise alignments between the pairs of CDS within each of the ten gene families of our dataset, yielding 4011 alignments in total for each of five methods. In the absence of available benchmarks for the direct evaluation of the accuracy of CDS alignments, we base our evaluation on four indirect strategies.

In the first strategy, we consider the CDS multiple alignment of each gene family obtained using MACSE [18] as a benchmark. This strategy exploits the fact that multiple alignments are usually more accurate than pairwise alignments. It then assumes that the MACSE multiple alignments are closer to the reality than the pairwise alignments obtained using the five methods. Note that all the pairwise alignment methods included in the comparison can be extended to multiple sequence alignment methods using classical strategies. Thus, the more accurate pairwise alignment methods should lead to more accurate multiple alignment methods. Here, we focus on the comparison of the pairwise versions of the methods. In the second strategy, we consider six composition criteria for a CDS pairwise alignment called Identity_NT, Identity_AA, Gap_init, Gap_length, FS_init, FS_length. The definitions of these criteria are given below, and used to compare the five methods. In the third strategy, we manually build and use as a benchmark, the real multiple alignment of three CDS from three paralogous human genes of the gene family I (FAM86). In the fourth strategy, we generate and use a set of three CDS splicing orthology groups, each group containing seven existing or putative CDS from seven genes of gene family I (FAM86).

Based on the results of the large-scale experiments discussed in the following, the best compromise for default values of FsePSA parameters are $-30$ for fs_open_cost and $-1$ for fs_extend_cost.

## Discussion

### First strategy: using MACSE multiple alignments as benchmark

MACSE [18] was used with its default parameters (fs_open_cost = $-30$, stop_cost = $-100$, $s_{aa} = \texttt {BLOSUM62 \quad matrix}$, gap_open_cost = $-7$, gap_cost = $-1$) to compute the CDS multiple alignment of each of the ten gene families. For each MACSE multiple alignment of *N* CDS, we consider the $\frac{N(N-1)}{2}$ induced pairwise alignments as a benchmark. In total, we then obtained a benchmark composed of 4011 pairwise alignments. In order to compare an alignment $(A',B')$ obtained with one of the five methods to the corresponding alignment $(A'',B'')$ in the benchmark, we computed the number of nucleotides aligned in $(A',B')$ with the same partner as in the benchmark alignment $(A'',B'')$.

Table 3 shows the overall percentage of nucleotides aligned with the same partners as in the benchmark for each of the compared methods, for varying fs_open_cost ($-10$, $-20$ and $-30$) and fs_extend_cost ($-1$, $-0.5$ and $-0.2$). It shows that the different versions of the fse method and the fse0 method have the best scores greater than $79.4\%$, followed by the needleprot method with a score of $78.82\%$. On the opposite, the needlenuc and macse_p method with fs_open_cost= $-30$ return the worst scores, respectively $50.95\%$ and $47.35\%$. These results also show that the fse method is more robust to the fs_open_cost parameter changes as compared to the macse_p method, whose scores show a large variation between 47.35 and 78.29%. Note that the needlenuc and needleprot do not account for the fs_open_cost parameter.

**Table 3:** Comparison with MACSE multiple alignments benchmark

fs_open_cost	fse0	fse (−1)	fse (−0.5)	fse (−0.2)	macse_p	needlenuc	needleprot
-10	*79.58 (1404)*	79.40 (1364)	*79.52 (1415)*	*79.58 (1433)*	77.17 (1076)	50.95 (255)	78.82 (972)
-20	*79.68 (1550)*	*79.68 (1526)*	*79.65 (1558)*	*79.67 (1552)*	78.29 (1389)		
-30	*79.75 (1558)*	*79.47 (1529)*	*79.60 (1546)*	*79.63 (1547)*	47.35 (742)		

Percentage of nucleotides aligned with the same partner as in the benchmark alignments induced by the MACSE multiple alignments, for each method for varying fs_open_cost ($-10$, $-20$ and $-30$) and fs_extend_cost ($-1$, $-0.5$ and $-0.2$). In each case, the number of CDS pairs with an alignment that presents the highest similarity with the corresponding benchmark alignment as compared to the other methods is given in parenthesis. The best results are indicated in italics

**Table 4:** Values of the six criteria for the *noFS* dataset (variations as compared to needleprot)

fs_open_cost (# CDS pairs)	Method	Identity_NT	Identity_AA	Gap_init	Gap_length	FS_init	FS_length
−10 (1672)	fse0	3281 (1158)	5376 (1222)	−495 (1606)	−2718 (1521)	0 (1672)	0 (1672)
	fse						
	macse_p	8120 (955)	27942 (676)	3701 (711)	9618 (1102)	0 (1672)	0 (1672)
	needlenuc	170239 (156)	−82002 (442)	104811 (218)	21422(427)	44488 (256)	263365 (256)
	*needleprot*	*1090957*	*2047608*	*10230*	*530688*	*0*	*0*
−20 (3441)	fse0	1409 (2612)	−8622 (2672)	−3564 (3169)	−9984 (3057)	0 (3441)	0 (3441)
	fse						
	macse_p	24909 (1437)	95844 (1011)	13778 (1076)	30884 (1791)	0 (3441)	0 (3441)
	needleenuc	547203 (176)	−177285 (680)	317256 (219)	52510 (552)	138204 (257)	844401 (257)
	*needleprot*	*2000228*	*3494760*	*31793*	*1313658*	*0*	*0*
−30 (3740)	fse0	1368 (2834)	−10788 (2912)	−4047 (3448)	−11316 (3321)	0 (3740)	0 (3740)
	fse						
	macse_p	27840 (1547)	106512 (1078)	15561 (1117)	34726 (1846)	0 (3740)	0 (3740)
	needlenuc	610305 (177)	−192231 (709)	351748 (219)	47356 (573)	154255 (257)	948418 (257)
	*needleprot*	* 2143630*	*3715632*	*35296*	*1439784*	* 0*	*0*

For varying values of the parameter fs_open_cost, the number of CDS pairs in the dataset is given.

The values of the criteria for the reference method “needleprot” are indicated in italics characters. For each of the other methods (fse, fse0, macse_p, needlenuc), the variations of the criteria values as compared to the reference values are given. For each criteria and each method, the number of CDS pairs that have the closest value to the reference needleprot value is given in parentheses

### Second strategy: using six composition criteria for CDS pairwise alignment

Six criteria were defined and used to compare the five pairwise alignment methods. Given a pairwise CDS alignment, the first criterion Identity_NT counts the number of gap-free columns in the alignment containing a nucleotide match. The second criterion Identity_AA counts the number of IM and FSext codons *c* in the alignment that are aligned with a triplet of nucleotides yielding the same amino acid as *c*. The third criterion Gap_init is the number of gap-containing columns in the alignment, either insertion or deletion columns that are preceded by a different type of column. The fourth criterion Gap_length is the overall number of gap-containing columns in the alignment. The fifth criterion FS_init is the number of FS translation segments found in the alignment. The last criterion FS_length is the overall number of columns in the alignment intersecting a FSext codon.

Note that the definitions of the six criteria exploit the definitions of codon sets used in Definition 3 but they are independent of any alignment scoring scheme. For example, for the alignment depicted in Fig. 2, $\texttt {Identity\_NT}=28$, counting all gap-free columns except the five columns at the positions $\{9,12,15,42,45\}$ containing a nucleotide mismatch. $\texttt {Identity\_AA}=14$, counting all IM and FSext codons except the two IM codons AAG and AAT ending at position 15 yielding two different amino acids *K* and *N*, and the FSext codon AAT ending at position 42 yielding the amino acid *N* different from the amino acid *K* yielded by the triplet AAG. $\texttt {Gap\_init} = 7$, counting the positions $\{4,16,22,27,31,36,44\}$. $\texttt {FS\_init} = 3$, counting the positions $\{18,28,39\}$. The two last criteria have the values $\texttt {Gap\_length} = 15$ and $\texttt {FS\_length} = 11$.

For each of the nine cases obtained by combining the values of the parameters fs_open_cost ($-10$, $-20$ or $-30$) and fs_extend_cost ($-1$, $-0.5$ or $-0.2$), we considered the 4011 pairs of CDS from the ten gene families dataset, and partitioned them into three sets. For each case, the first set called the noFS dataset is composed of the pairs of CDS for which the pairwise alignments obtained using the fse0, fse and macse_p methods all have the criteria $\texttt {FS\_init} = 0$. The second set called the FS dataset is composed of the pairs of CDS for which the alignments obtained using the fse0, fse and macse_p methods all have the criteria $\texttt {FS\_init} > 0$. The third set called the ambiguFS dataset is composed of the remaining pairs of CDS.

Note that, in all nine cases, the set of CDS pairs for which $\texttt {FS\_init} = 0$ with the macse_p method was strictly included in the set of CDS pairs for which $\texttt {FS\_init} = 0$ with the fse method. For each of the nine cases, we computed the overall value of the six criteria for each method (fse0, fse, macse_p, needlenuc and needleprot) and each dataset (noFS, FS and ambiguFS). Tables 4, 5 and 6 present the results.

#### Results for the noFS datasets

For the noFS datasets, we assume that the real alignments should not contain FS translations. So, the needleprot method most likely computes the more accurate alignments since it does not allow any FS translation in the alignments. Indeed, it computes a maximum score NW alignment at the AA level and back-translates this alignment at the nucleotide level. We then take the needleprot result as a reference for the noFS dataset, in all cases. By construction of the noFS dataset, for a fixed value of the parameter fs_open_cost, the fse0 and fse methods necessarily return two alignments with the same similarity score for each pair of CDS of the dataset. Indeed, we observed that, for each value of fs_open_cost ($-10$, $-20$ or $-30$), the alignments obtained using the methods fse0 or fse with varying values of the parameter fs_extend_cost are unchanged.

Table 4 summarizes the results for $\texttt {fs\_open\_cost}=-10$, $-20$ and $-30$, presenting the results of the varying versions of fse and fse0 in a single line in the three cases. It shows that the results of the fse and fse0 methods are the closest to the reference for all the six criteria in all cases. However, they slightly overestimate or underestimate the criteria. The tendency of overestimating the Identity_AA and all other criteria is particularly accentuated for the macse_p method as compared to the fse and fse0 methods, in all cases. On the opposite, the needlenuc method always largely underestimates the Identity_AA, while overestimating all other criterion.

#### Results for the FS datasets

For the FS datasets, we assume that the real alignments must contain FS translations. So, the needleprot method can no longer produce the most accurate results. On the contrary, it is most likely that it underestimates the Identity_AA criterion. Indeed, it correctly aligns AA in CDS regions that are free of FS translation, but in FS translation regions, it either leads to several AA mismatches in the case of high mismatches scores, or to an overestimation of the Gap_init criterion. As expected, we observed that the value of Identity_AA for the needleprot method was always the lowest (data shown in the Additional file 3). We focus on the four other methods.

Table 5 summarizes the results for the nine cases considered. For the Identity_NT and Identity_AA criteria, the differences between the values for the four methods are negligible. The main differences between the results reside in the values of the Gap_init and FS_init criteria. In particular, the FS_init criterion is useful to compare the accuracy of the methods for correctly identifying real FS translation regions. In [6] (for family I) and [12] (for families II–X), at most one FS translation region was detected and manually validated for each pair of CDS of the ten gene families. So, the expected number of FS translation regions per alignment in the FS data is 1. In Table 5, we observe that, in all cases, the fse and fse0 methods are the only methods for which the average numbers of FS_init are close to 1 with +/− standard error values smaller than 1. The macse method and especially the needlenuc method overestimate the number of FS translation regions per alignment with large standard error values in all cases.

#### Results for the ambiguFS datasets

For the ambiguFS datasets, all methods do not agree for the presence or absence of FS translation regions between the pairs of CDS. Note that the needlenuc method reports FS translations for all pairs of CDS, with the highest average number of FS translation regions per alignment in all cases (data shown in the Additional file 3). As needlenuc is already shown to perform poorly in both the absence and the presence of FS translation regions, we focus on the four other methods. Table 6 summarizes the results. We observe that, for all criteria, macse_p has higher values than fse0, fse and needleprot that have similar values. The most significant difference between the results resides in the values for the FS_init and FS_length criteria. The fse method always reports a null or a very small number of FS regions with an average FS_init equals to 1 as expected. In all cases, the fse0 and macse methods overestimate the number of FS translation regions per alignment.

**Table 5:** Values of the six criteria for the* FS* dataset

fs_open_ cost	fs_extend_cost (# CDS pairs)	Method	Identity_ NT	Identity_ AA	Gap_ init	Gap_ length	FS_init (avg)	FS_ length
−10	−1 (212)	*fse0*	166002	325212	895	60662	226 (*1.06 ± 0.25*)	20219
		*fse*	165720	325026	901	60624	216 (*1.01* *± 0.14*)	18705
		macse_p	166167	324999	1445	61562	432 (2.03 ± 3.06)	22742
		needlenuc	172959	321348	5053	60038	2103 (9.91 ± 26.73)	29616
	−0.5 (386)	*fse0*	252590	464712	2400	114859	482 (*1.24 ± 0.47*)	31777
		*fse*	251647	463407	2387	115269	401 (*1.03* *± 0.1*9)	26982
		macse_p	253715	465594	4161	117165	1306 (3.38 ± 4.53)	41742
		needlenuc	279682	452673	19408	113195	8032 (20.80 ± 31.02)	68226
	−0.2 (619)	*fse0*	371062	641748	5334	204370	805 (*1.30 ± 0.52*)	43381
		*fse*	370260	640377	5270	204806	688 (*1.11* * ± 0.33*)	37376
		macse_p	374729	646893	9308	208344	2893 (4.67 ± 5.34)	72030
		needlenuc	442564	618270	48799	209420	19751 (31.90 ± 34.48)	141217
−20	−1 (161)	*fse0*	123814	244350	461	40315	168 (*1.04 ± 0.20*)	17770
		*fse*	123610	244149	468	40195	164 (*1.01* *± 0.14*)	16924
		macse_p	123541	243591	709	40585	223 (1.38 ± 1.03)	18119
		needlenuc	125452	242742	1493	39031	650 (4.03 ± 5.85)	19405
	−0.5 (189)	*fse0*	147476	291147	549	49485	197 (*1.04 ± 0.20*)	19599
		*fse*	147401	291048	557	49363	194 (*1.02* *± 0.16* )	19279
		macse_p	147143	290271	838	49841	260 (1.37 ± 0.98)	19976
		needlenuc	149551	289086	1872	47515	808 (4.27 ± 6.17)	21440
	−0.2 (216)	*fse0*	161906	318117	723	55383	225 (*1.04 ± 0.20*)	21300
		*fse*	161865	318099	732	55393	223 ( *1.03 ±* * 0.18*)	21115
		macse_p	161622	317205	1061	55715	306 (1.41 ± 0.99)	21997
		needlenuc	165260	315531	2851	53613	1186 (5.49 ± 6.82)	24403
−30	−1 (71)	*fse0*	47071	91266	230	26303	76 (*1.07 ± 0.26*)	12845
		*fse*	46872	91032	233	26183	72 (*1.01* *± 0.12*)	12302
		macse_p	46936	90876	372	26325	118 (1.66 ± 1.25)	13142
		needlenuc	48290	91017	866	26135	391 (5.50 ± 5.67)	13829
	−0.5 (154)	*fse0*	120558	237768	445	37975	159 (*1.03 ± 0.18*)	17554
		*fse*	120504	237678	452	37851	157 (*1.01 ±* * 0.14*)	17319
		macse_p	120338	237084	691	38047	212 (1.37 ± 1.00)	17926
		needlenuc	122084	236904	1321	37531	575 (3.73 ± 5.14)	18877
	−0.2 (178)	*fse0*	137451	271041	525	46049	184 (*1.03 ± 0.18*)	18995
		*fse*	137440	271008	531	45917	183 (*1.02* *± 0.17*)	18872
		macse_p	137175	270258	803	46187	244 ( 1.37 ± 0.97)	19395
		needlenuc	139489	269139	1803	44303	779 (4.38 ± 6.27)	20859

For varying values of the parameters fs_open_cost and fs_extend_cost, the number of CDS pairs in the dataset is given. The values of the criteria for the fse, fse0, macse_p, needlenuc methods are indicated. For each method, the average number of FS_init per alignment, with corresponding standard error values are also indicated

The best results are indicated in italics

**Table 6:** Values of the six criteria for the *ambiguFS* dataset

fs_open_ cost	fs_extend_cost (# CDS pairs)	Method	Identity_ NT	Identity_ AA	Gap_ init	Gap_ length	FS_init (avg)	FS_ length
−10	−1 (2127)	fse0 (862)	1095102	1737105	24489	908218	1111 (1.28 $\pm\,0.54$)	42730
		fse	1086546	1719774	23483	906540	0	0
		macse_p (2076)	1124316	1790199	45335	936002	12436 (5.99 $\pm\,4.96$)	216772
		needleprot	1085007	1723950	25288	916518	0	0
	−0.5 (1953)	fse0 (688)	1008514	1597605	22984	854021	855 (1.24 $\pm\,0.53$ )	31172
		fse (2)	1003293	1587258	22102	853793	2 (1.0 $\pm\,0$ )	80
		macse_p (1902)	1036768	1649604	42619	880399	11562 (6.07 $\pm\,4.91$)	197772
		needleprot	1001957	1591134	23790	863199	0	0
	−0.2 (1720)	fse0 (455)	890042	1420569	20050	764510	532 (1.16 $\pm\,0.48$)	19568
		fse (3)	887372	1415403	19465	764162	3 (1.0 $\pm\,0$)	92
		macse_p (1669)	915754	1468305	37472	789220	9975 (5.97 $\pm\,4.75$)	167484
		needleprot	886178	1418748	20955	772272	0	0
−20	−1 (409)	fse0 (100)	219277	358554	3633	153487	120 (1.2 $\pm\,0.40$)	6937
		fse	216936	353586	3619	152391	0	0
		macse_p (403)	225976	374391	6509	158165	1348 (3.34 $\pm\,3.00$)	36179
		needleprot	216842	355656	4172	153957	0	0
	−0.5 (381)	fse0 (72)	195615	311757	3545	144317	91 (1.26 $\pm\,0.44$)	5108
		fse	194048	308448	3505	144045	0	0
		macse_p (375)	202374	327711	6380	148909	1311 (3.49 $\pm\,3.05$)	34322
		needleprot	193980	310632	4051	145563	0	0
	−0.2 (354)	fse0 (45)	181185	284787	3371	138419	63 (1.4 $\pm\,0.49$)	3407
		fse (1)	180151	282693	3344	138217	1 (1.0 $\pm\,0$)	40
		macse_p (348)	187895	300777	6157	143035	1265 (3.63 $\pm\,3.11$)	32301
		needleprot	180116	284946	3883	139731	0	0
−30	−1 (200)	fse0 (119)	151090	289617	805	42437	120 (1.01 $\pm\,0.09$)	6818
		fse	147590	282018	852	40221	0	0
		macse_p (200)	152626	292254	1309	43043	378 (1.89 $\pm\,2.16$)	14515
		needleprot	147228	281472	933	40455	0	0
	−0.5 (117)	fse0 (36)	77603	143115	590	30765	37 (1.02 $\pm\,0.16$)	2109
		fse	76678	141108	626	29913	0	0
		macse_p (117)	79224	146046	990	31321	284 (2.42 $\pm\,2.65$)	9731
		needleprot	76561	141036	703	30099	0	0
	−0.2 (93)	fse0 (12)	60710	109842	510	22691	12 (1.0 $\pm\,0$)	668
		fse	60407	109170	518	22491	0	0
		macse_p (93)	62387	112872	878	23181	252 (2.70 $\pm\,2.89$)	8262
		needleprot	60270	109122	581	22677	0	0

For varying values of the parameters fs_open_cost and fs_extend_cost, and for each method, the number of CDS pairs displaying a FS translation is given. The values of the criteria for each method are indicated. For each method, the average number of FS_init per alignment, with corresponding standard error values are also indicated

### Third strategy: using a 3-CDS manually-built benchmark

We manually built the real pairwise alignments of three CDS from three paralogous human genes of gene family I, the CDS *FAM86C1-002* coding for protein *ENSP00000352182.4*, *FAM86B1-001* coding for protein *ENSP00000431362.1* and *FAM86B2-202* coding for protein *ENSP00000311330.6*. The real multiple alignment of the three CDS is roughly depicted and detailed in Fig. 4. From Fig. 4, we observe that *FAM86C1-002* shares with *FAM86B1-001* a nucleotide region of length 159 translated in the same frame and a nucleotide region of length 89 with FS translation, while it only shares with *FAM86B2-202* a nucleotide region of length 238 ($89+149$) entirely under FS translation. It is then clear that CDS *FAM86C1-002* and *FAM86B1-001* are the most similar. Figure 4 also shows that each pair of CDS shares a single FS translation region.

Table 7 shows the normalized pairwise similarity scores and the number of FS translation regions computed by the five alignment methods (the pairwise alignments computed by the five methods with varying fs_open_cost and fs_extend_cost are given in the Additional file 4). It shows that needleprot and fse (in all cases where fs_extend_cost= −1) are the only two methods that allow to infer that *FAM86C1-002* and *FAM86B1-001* are the most similar. Table 7 also illustrates the fact that needlenuc and macse_p strongly overestimate the number of FS translation regions per alignment in all cases. The fse method with the parameters fs_open_cost= −10 and fs_extend_cost= −1 is the only method that allows to infer that *FAM86C1-002* and *FAM86B1-001* are the most similar and to detect a single FS translation region for each alignment.

**Table 7:** Pairwise similarity scores and number of FS translation regions computed by the methods

fs_open_cost	Method	*C1-002* vs *B1-001*	*C1-002* vs *B2-202*	*B1-001* vs *B2-202*
−10	fse0	0.42 (1)	0.58 (2)	0.45 (1)
	fse (-1)	0.33 (1)	0.27 (1)	0.18 (1)
	fse (-0.5)	0.37 (1)	0.43 (1)	0.31 (1)
	fse (-0.2)	0.40 (1)	0.52 (1)	0.39 (1)
	macse_p	0.40 (4)	0.54 (6)	0.44 (1)
−20	fse0	0.39 (1)	0.54 (1)	0.41 (1)
	fse (-1)	0.36 (0)	0.24 (1)	0.14 (1)
	fse (-0.5)	0.34 (1)	0.39 (1)	0.28 (1)
	fse (-0.2)	0.37 (1)	0.48 (1)	0.36 (1)
	macse_p	0.33 (4)	0.47 (6)	0.35 (1)
−30	fse0	0.35 (1)	0.50 (1)	0.38 (1)
	fse (-1)	0.36 (0)	0.20 (1)	0.11 (1)
	fse (-0.5)	0.36 (0)	0.35 (1)	0.25 (1)
	fse (-0.2)	0.33 (1)	0.44 (1)	0.33 (1)
	macse_p	0.27 (4)	0.39 (6)	0.29 (1)
	needlenuc	0.16 (23)	0.35 (15)	−0.36 (1)
	needleprot	0.38 (0)	−0.12 (0)	−0.13 (0)

Normalized pairwise similarity scores and number of FS translation regions computed by the five methods for the 3-CDS manually-built benchmark composed of CDS *FAM86C1-002*, *FAM86B1-001* and *FAM86B2-202* (Similarity scores are normalized by dividing them by the lengths of alignments)

### Fourth strategy: inferring CDS splicing orthology groups and protein phylogenies

Based on the three CDS used in the previous strategy, CDS *FAM86C1-002* from human gene *ENSG00000158483*, *FAM86B1-001* from human gene *ENSG00000186523* and *FAM86B2-202* from human gene *ENSG00000145002*, we generated a dataset of three CDS splicing orthology groups composed of 21 homologous CDS. Each group contains one of the three initial CDS and its six splicing orthologs in the following set of seven genes from gene family I: human genes *ENSG00000158483* denoted *H*1, *ENSG00000186523* denoted *H*2 and *ENSG00000145002* denoted *H*3, each containing one of the initial CDS, chimpanzee gene *ENSPTRG00000007738* denoted *Ch*, mouse gene *ENSMUSG00000022544* denoted *M*, rat gene *ENSRNOG0-0000002876* denoted *R* and cow gene *ENSBTAG00000008222* denoted *Co*. The CDS splicing orthologs were predicted based on the spliced alignment tool Splign [28] as follows: for each initial CDS $A_1$ of a gene *A* and each gene *B* different from *A*, $A_1$ was aligned to *B* and a putative or existing CDS of *B* ortholog to $A_1$ with the same splicing structure was inferred. The 21 resulting CDS are given in Additional file 5.

We computed the normalized pairwise similarity scores between the CDS, using the five alignment methods (the pairwise alignments computed by the five methods with varying fs_open_cost and fs_extend_cost are given in the Additional file 5). For each method, we constructed a phylogeny using an UPGMA and a Neighbor-Joining (NJ) algorithm, based on the computed CDS similarity matrix. The UPGMA algorithm was used to classify the CDS into three groups and infer the similarity relationships between the groups independently of any rate of evolution. The NJ algorithm was used to reconstruct the phylogeny inside each group. Table 8 summarizes the results. The three splicing orthology groups are denoted *G1* (containing CDS *C1-002*), *G2* (containing CDS *B1-001*) and *G3* (containing CDS *B2-202*).

All methods allow to correctly classify the CDS into the three initial splicing orthology groups G1, G2, and G3. However, the needleprot and fse methods are the only methods that allow to infer the correct similarity relationships ((G1,G2),G3) between the groups, confirming the results of the third evaluation strategy. For all methods, the CDS phylogeny reconstructed inside the group G2 is (Co,((M,R),((H1,Ch),(H2,H3)))) inducing an evolution of the seven genes with a speciation event at the root of the gene tree. The phylogeny reconstructed for the groups G1 and G3 is ((M,R),(Co,((H1,Ch),(H2,H3)))), inducing an evolution of the genes with a duplication event at the root of the phylogeny.

**Table 8:** Similarity relationships between the groups G1, G2 and G3 for the five methods

fs_open_cost	Method	((G1,G3),G2)	((G1,G2),G3)
−10	fse (-1)		X
	fse (-0.5)		X
	fse (-0.2)	X	
	fse0	X	
	macse_p	X	
−20	fse (-1)		X
	fse (-0.5)		X
	fse (-0.2)		X
	fse0	X	
	macse_p	X	
−30	fse (-1)		X
	fse (-0.5)		X
	fse (-0.2)		X
	fse0		X
	macse_p	X	
	needlenuc	X	
	needleprot		X

Similarity relationships between the splicing orthology groups G1, G2 and G3 computed using the similarity matrices of the five methods for the 21-CDS dataset

### Comparing of the running times

Table 9 shows the running times for each of the five methods on the three first gene families of our dataset on a $24 \times 2.1$ GHz processor with 10 GB of RAM. The needleprot method is the fastest, followed by macse_p and then needlenuc, while fse and fse0 are the slowest methods.

Note that for fse, fse0, needlenuc and needleprot, the used implementations are in Python, while we used a JAVA implementation for macse_p provided by its authors. This explains the fact that macse_p is unexpectedly faster here than fse, fse0, and even needlenuc. Indeed, the five methods share the same asymptotic time complexity, but the exact complexity of each of them is dependent on the number of calls of the main recurrence formulas in an execution, and the number of cases considered in each recurrence formula. The exact computational complexity of the five methods in terms of the lengths *n* and *m* of two compared CDS are 12.55× nm for fse and fse0 (as shown in the proof of Theorem 1), 3× nm for macse_p, 0.33× nm for needlenuc and 0.33× nm for needleprot.

**Table 9:** Running time in seconds for each method

Gene family	fse0	fse	macse_p	needlenuc	needleprot
I	299	291	53	97	22
II	270	260	45	93	20
III	377	389	54	62	20

For each method and gene families I, II, and III, the running time was calculated on the same computer (24 processors of 2.1GHz each and 10GB of RAM) with the parameters fs_open_cost = $-20$ and fs_extend_cost = $-0.2$

## Conclusions

In this paper, we introduce a new scoring model for the alignment of CDS accounting for frameshift translation length. The motivation for this new scoring scheme is the increasing evidence for protein divergence through frameshift translation in eukaryotic coding gene families, calling for automatic methods able to compare, align and classify CDS while accounting for their codon structure. The aim of this paper is to validate the necessity of accounting for frameshift translation length when comparing CDS and show that computing a maximum score pairwise alignment under the new scoring scheme is possible in quadratic time complexity. The results of comparing five CDS alignment methods for the pairwise alignment of CDS from ten eukaryotic gene families show that our method is the best compromise for sets of CDS in which some pairs of CDS display FS translations while some do not. Future work will make use of benchmarks of CDS alignments generated manually and by simulation in order to confirm these experimental results. We also defer to a future work the extended study of our model’s robustness to parameter changes and the calibration of its parameters using real data benchmarks. The perspectives of this work also include the design of a heuristic algorithm using local alignment that will achieve scalability for large datasets while keeping high accuracy, and the extension of the method toward multiple alignment. Finally, we plan to apply the algorithms for the discovery of non-annotated frameshifts, and the evaluation of the extent of frameshifts in eukaryotic gene families.

## Additional files

**Additional file 1:** Proof of Lemma 1. File containg the detailed proof of Lemma 1.

**Additional file 2:** CDS of the ten gene families. Zip file containing the CDS files at the fasta format for each of the ten gene families considered in the “Results” section.

**Additional file 3:** Additional lines for Tables 5 and 6. File containing additional lines for Tables 5 (for needleprot) and 6 (for needlenuc) of the “Results” section.

**Additional file 4:** Pairwise alignments for the 3-CDS benchmark. Zip file containing the sequence file and the pairwise alignment files at the fasta format for the manually-built3-CDS benchmark considered in the “Results” section, for each of the five methods and each parameter configuration.

**Additional file 5:** Pairwise alignments for the 21-CDS dataset. Zip file containing the sequence file and the pairwise alignment files at the fasta format for the 21-CDS benchmarkconsidered in the “Results” section, for each of the five methods and each parameter configuration.

## Abbreviations

CDS: coding DNA sequence
FS: frameshift
NT: nucleotide
AA: amino acid
NW: Needleman–Wunsch
