# Supplementary material for: Aligning coding sequences with frameshift extension penalties
Source: Algorithms Mol Biol. 2017 Mar 31;12:10. doi: 10.1186/s13015-017-0101-4 (PMC5374649; doi:10.1186/s13015-017-0101-4)
Supplement: Supplementary file 1 — Additional file 1: Proof of Lemma 1. File containg the detailed proof of Lemma 1. [file 13015_2017_101_MOESM1_ESM.pdf]

Additional file 1 – Proof of Lemma1
Aligning coding sequences with frameshift extension penalties

Safa Jammali et al.
Department of Computer Science, Université de Sherbrooke,
Sherbrooke, QC, Canada
Email: safa.jammali@usherbrooke.ca

Following the definitions and notations for frameshifts used in [14], given an alignment between two CDS A and B, the set of `FSinit` codons of A (resp. B) can be divided into two sets. The set of `FSinit codons caused by deletions (FS`$^-$`)` contains the `FSinit` codons that are grouped in the alignment and are aligned with at least one nucleotide and at least one gap symbol in the other CDS. The set of `FSinit codons caused by insertions (FS`$^+$`)` contains all the codons that are not grouped in the alignment.

Proof of Lemma 1

The principle of the proof is similar to the one for the alignment of non-coding sequences. For each of the cases 1, 2, 3 and 4 of the Lemma, the score $D(i, j)$ is the maximum score of all possible alignment configurations that are considered for this case. An illustration of the different configurations of alignment considered for the cases 1 and 2 is shown in Figure 2 of the main manuscript.

For each case, we first consider three sub-cases depending on the configurations of the alignment of $A[i]$ and $B[j]$: (a) $A[i]$ and $B[j]$ are aligned together, (b) $A[i]$ is aligned with a gap, (c) $B[j]$ is aligned with a gap.

1. **If** $i(mod\ 3) = 0$ **and** $j(mod\ 3) = 0$, then $A[i]$ and $B[j]$ are the last nucleotides of two codons $A[i-2\ ..\ i]$ and $B[j-2\ ..\ j]$. There are three cases depending on the alignment of $A[i]$ and $B[j]$.

   (a) **If** $A[i]$ **and** $B[j]$ **are aligned together**, there are four cases depending on whether $A[i-2\ ..\ i]$ and $B[j-2\ ..\ j]$ are grouped in the alignment or not.

      i. **If both** $A[i-2\ ..\ i]$ **and** $B[j-2\ ..\ j]$ **are grouped**, then $A[i-2\ ..\ i]$ and $B[j-2\ ..\ j]$ have to be aligned together and

the score of the alignment is:

1. $s_{aa}(A[i-2\ ..\ i], B[j-2\ ..\ j]) + D(i-3, j-3)$

ii. **If** $A[i-2\ ..\ i]$ **is grouped while** $B[j-2\ ..\ j]$ **is not grouped**, then both $A[i-2\ ..\ i]$ and $B[j-2\ ..\ j]$ are FS codons ($A[i-2\ ..\ i]$ is a FS^- codon while $B[j-2\ ..\ j]$ is a FS^+ codon). We add $2 * \texttt{fs\_open\_cost}$ to the score of the alignment and the alignment of the nucleotides of the two FS codons can be scored independently using the scoring function s_{an}. There are two cases depending on the number of nucleotides from $B[j-2\ ..\ j]$ that are aligned with $A[i-2\ ..\ i]$, two or one:

A. **If** $A[i-2\ ..\ i]$ **is aligned with two nucleotides**, then these nucleotides are $B[j-1]$ and $B[j]$. There are two cases depending on the alignment of the nucleotide $B[j-1]$ with $A[i-1]$ or $A[i-2]$:

2. $s_{an}(A[i], B[j]) + s_{an}(A[i-1], B[j-1]) + D(i-3, j-2) + 2 * \texttt{fs\_open\_cost}$

3. $s_{an}(A[i], B[j]) + s_{an}(A[i-2], B[j-1]) + D(i-3, j-2) + 2 * \texttt{fs\_open\_cost}$

B. **If** $A[i-2\ ..\ i]$ **is aligned with one nucleotide**, then this single nucleotide is $B[j]$ and the score of the alignment is:

4. $s_{an}(A[i], B[j]) + D(i-3, j-1) + 2 * \texttt{fs\_open\_cost}$

iii. **If** $A[i-2\ ..\ i]$ **is not grouped while** $B[j-2\ ..\ j]$ **is grouped**, there are three cases that are symmetric to the three cases from (a)ii.:

5. $s_{an}(A[i], B[j]) + s_{an}(A[i-1], B[j-1]) + D(i-2, j-3) + 2 * \texttt{fs\_open\_cost}$

6. $s_{an}(A[i], B[j]) + s_{an}(A[i-1], B[j-2]) + D(i-2, j-3) + 2 * \texttt{fs\_open\_cost}$

7. $s_{an}(A[i], B[j]) + D(i-1, j-3) + 2 * \texttt{fs\_open\_cost}$

iv. **If both** $A[i-2\ ..\ i]$ **and** $B[j-2\ ..\ j]$ **are not grouped**, then again both $A[i-2\ ..\ i]$ and $B[j-2\ ..\ j]$ are FS codons (both are FS^+ codons):

8. $s_{an}(A[i], B[j]) + D(i-1, j-1) + 2 * \texttt{fs\_open\_cost}$

(b) **If** $A[i]$ **is aligned with a gap**, then the codon $A[i-2\ ..\ i]$ is a FS codon (FS^- or FS^+). We must add `fs_open_cost` to the score of the alignment. There are two cases depending on whether $A[i-2\ ..\ i]$ is grouped in the alignment or not.

i. **If** $A[i-2\ ..\ i]$ **is grouped**, then there are three cases depending on the number of nucleotides from $B[j-2\ ..\ j]$ that are aligned with $A[i-2\ ..\ i]$, two, one, or zero.

A. **If $A[i-2 .. i]$ is aligned with two nucleotides**, then these nucleotides are $B[j-1]$ and $B[j]$. The score of the alignment is:
9. $\frac{s_{an}(A[i-1],B[j])}{2} + \frac{s_{an}(A[i-2],B[j-1])}{2} + D_F(i-3, j-2) +$ `fs_open_cost`

B. **If $A[i-2 .. i]$ is aligned with one nucleotide**, then this single nucleotide is $B[j]$. There two cases depending on the alignment of the nucleotide $B[j]$ with $A[i-1]$ or $A[i-2]$:
10. $s_{an}(A[i-1], B[j]) + D(i-3, j-1) + 2 *$ `fs_open_cost`
11. $\frac{s_{an}(A[i-2],B[j])}{2} + D_F(i-3, j-1) +$ `fs_open_cost`

C. **If $A[i-2 .. i]$ is aligned with zero nucleotide**, then the codon $A[i-2 .. i]$ is entirely deleted. The score of the alignment is:
12. `gap_cost` $+ D(i-3, j)$

ii. **If $A[i-2 .. i]$ is not grouped**, then the codon $A[i-2 .. i]$ is a FS^+ codon and the score of the alignment is:
13. $D(i-1, j) +$ `fs_open_cost`

(c) **If $B[i]$ is aligned with a gap**, there are fives cases that are symmetric to the five cases from (b):
14. $\frac{s_{an}(A[i],B[j-1])}{2} + \frac{s_{an}(A[i-1],B[j-2])}{2} + D_F(i-2, j-3) +$ `fs_open_cost`
15. $s_{an}(A[i], B[j-1]) + D(i-1, j-3) + 2 *$ `fs_open_cost`
16. $\frac{s_{an}(A[i],B[j-2])}{2} + D_F(i-1, j-3) +$ `fs_open_cost`
17. `gap_cost` $+ D(i, j-3)$
18. $D(i, j-1) +$ `fs_open_cost`

2. **If $i(mod\ 3) = 0$ and $j(mod\ 3) \neq 0$**, then $A[i]$ is the last nucleotide of a codon $A[i-2 .. i]$ and $B[j]$ is not the last nucleotide of a codon. There are three cases depending on the alignment of $A[i]$ and $B[j]$.

(a) **If $A[i]$ and $B[j]$ are aligned together**, there are two cases depending on whether $A[i-2 .. i]$ is grouped in the alignment or not.

i. **If $A[i-2 .. i]$ is grouped**, there are three cases depending on the number of nucleotides from B that are aligned with $A[i-2 .. i]$, three, two, or one:

A. **If $A[i-2 .. i]$ is aligned with three nucleotides**, then these nucleotides are $B[j]$, $B[j-1]$ and $B[j-2]$. We are in the case of a FSext codon. The score of the alignment is then:

1. $\frac{s_{aa}(A[i-2\ ..\ i],B[j-2\ ..\ j])}{2} + D_F(i-3, j-3) + \texttt{fs\_extension\_cost} + \frac{s_{an}(A[i],B[j])}{2}\ (+\frac{s_{an}(A[i-1],B[j-1])}{2}\ if\ j-1(mod\ 3) \neq 0)$

B. **If** $A[i-2\ ..\ i]$ **is aligned with two nucleotides**, then these nucleotides are $B[j]$ and $B[j-1]$. $A[i-2\ ..\ i]$ is a FS$^-$ codon. There are two cases depending of the alignment of $B[j-1]$ with $A[i-1]$ or $A[i-2]$. In both cases, if $j-1(mod\ 3) = 0$, then $j-1$ is the last nucleotide of a codon. We should then make adjustments in order to account for the type of this codon (FS$^+$, or unknown type for now):

2. $s_{an}(A[i], B[j]) + s_{an}(A[i-1], B[j-1]) + D(i-3, j-2) + \texttt{fs\_open\_cost}\ (+\texttt{fs\_open\_cost}\ if\ j-1(mod\ 3) = 0)$

3. $s_{an}(A[i], B[j]) + s_{an}(A[i-2], B[j-1]) + D_F(i-3, j-2) + \texttt{fs\_open\_cost}\ (-\frac{s_{an}(A[i-2],B[j-1])}{2}\ if\ j-1(mod\ 3) = 0)$

C. **If** $A[i-2\ ..\ i]$ **is aligned with one nucleotide**, then $A[i-2\ ..\ i]$ is a FS$^-$ codon. The score of the alignment is:

4. $s_{an}(A[i], B[j]) + D(i-3, j-1) + \texttt{fs\_open\_cost}$

ii. **If** $A[i-2\ ..\ i]$ **is not grouped**, then $A[i-2\ ..\ i]$ is a FS$^+$ codon:

5. $s_{an}(A[i], B[j]) + D(i-1, j-1) + \texttt{fs\_open\_cost}$

(b) **If** $A[i]$ **is aligned with a gap**, there are two cases depending on whether $A[i-2\ ..\ i]$ is grouped in the alignment or not.

i. **If** $A[i-2\ ..\ i]$ **is grouped**, there are three cases depending on the number of nucleotides from B that are aligned with $A[i-2\ ..\ i]$, two, one, or zero.

A. **If** $A[i-2\ ..\ i]$ **is aligned with two nucleotides**, then these nucleotides are $B[j]$ and $B[j-1]$. $A[i-2\ ..\ i]$ is a FS$^-$ codon. If $j-1(mod\ 3) = 0$, then $j-1$ is the last nucleotide of a codon. We should make adjustments in order to account for the fact no type has yet been decided for this codon.

6. $s_{an}(A[i-1], B[j]) + s_{an}(A[i-2], B[j-1]) + D_F(i-3, j-2) + \texttt{fs\_open\_cost}\ (-\frac{s_{an}(A[i-2],B[j-1])}{2}\ if\ j-1(mod\ 3) = 0)$

B. **If** $A[i-2\ ..\ i]$ **is aligned with one nucleotide**, then this single nucleotide is $B[j]$. $A[i-2\ ..\ i]$ is a FS$^-$ codon. There are two cases depending on the alignment of $B[j]$ with $A[i-1]$ or $A[i-2]$:

7. $s_{an}(A[i-1], B[j]) + D(i-3, j-1) + \texttt{fs\_open\_cost}$

8. $s_{an}(A[i-2], B[j]) + D(i-3, j-1) + \texttt{fs\_open\_cost}$

C. **If** $A[i-2\ ..\ i]$ **is aligned with zero nucleotide**, the

codon $A[i-2\ ..\ i]$ is entirely deleted:
9. $\texttt{gap\_cost} + D(i-3, j)$

ii. **If** $A[i-2\ ..\ i]$ **is not grouped**
10. $D(i-1, j) + \texttt{fs\_open\_cost}$

(c) **If** $B[j]$ **is aligned with a gap**, then the score of the alignment is:
11. $D(i, j-1)$

3. **If** $i(mod\ 3) \neq 0$ **and** $j(mod\ 3) = 0$, the proof is symmetric to the previous proof for 2.

4. **If** $i(mod\ 3) \neq 0$ **and** $j(mod\ 3) \neq 0$, there are three cases depending on the alignment of $A[i]$ and $B[j]$.

   (a) **If** $A[i]$ **and** $B[j]$ **are aligned together**, the score of the alignment is:
   1. $s_{an}(A[i], B[j]) + D(i-1, j-1)$

   (b) **If** $A[i]$ **is aligned with a gap**, the score of the alignment is:
   2. $D(i-1, j)$

   (c) **If** $B[j]$ **is aligned with a gap**, the score of the alignment is:
   3. $D(i, j-1)$

□
