# Supplementary material for: Aligning coding sequences with frameshift extension penalties
Source: Algorithms Mol Biol. 2017 Mar 31;12:10. doi: 10.1186/s13015-017-0101-4 (PMC5374649; doi:10.1186/s13015-017-0101-4)
Supplement: Supplementary file 3 — Additional file 3: Additional lines for Tables 5 and 6. File containing additional lines for Tables 5 (for needleprot) and 6 (for needlenuc) of the “Results” section. [file 13015_2017_101_MOESM3_ESM.pdf]

Additional file 3 – Additional lines for Tables 5 and 6
Aligning coding sequences with frameshift extension penalties

Safa Jammali et al.
Department of Computer Science, Université de Sherbrooke,
Sherbrooke, QC, Canada
Email: safa.jammali@usherbrooke.ca

Values of the six composition criteria for the `needleprot` method on the `FS` dataset (complement of Table 5 in the main manuscript).

fs_open_cost	fs_extend_cost (# CDS pairs)	Identity_NT	Identity_AA	Gap_open	Gap_length	FS_init	FS_length
-10	-1 (212)	154976	301704	1295	59328	0	0
	-0.5 (386)	238026	434520	2793	112647	0	0
	-0.2 (619)	353805	606906	5628	203574	0	0
-20	-1 (161)	113870	222846	848	38919	0	0
	-0.5 (189)	136732	267870	969	47313	0	0
	-0.2 (216)	150596	293556	1137	53145	0	0
-30	-1 (71)	40082	76158	584	26295	0	0
	-0.5 (154)	110749	216594	814	36651	0	0
	-0.2 (178)	127040	248508	936	44073	0	0

For varying values of the parameters `fs_open_cost` and `fs_extend_cost`, the values of the criteria for the `needleprot` method.

Values of the six composition criteria for the `needlenuc` method on the `ambiguFS` dataset (complement of Table 6 in the main manuscript).

fs_open_cost	fs_extend_cost (# CDS pairs)	Identity_NT	Identity_AA	Gap_open	Gap_length	FS_init	FS_length
-10	-1 (2127)	1527999	1617300	273104	943464	110296 (51.85)	689967
	-0.5 (1953)	1421276	1485975	258749	890307	104367 (53.43)	651357
	-0.2 (1720)	1258394	1320378	229358	794082	92648 (53.86)	578366
-20	-1 (409)	289271	344037	42656	150413	18033 (44.09)	119142
	-0.5 (381)	265172	297693	42277	141929	17875 (46.91)	117107
	-0.2 (354)	249463	271248	41298	135831	17497 (49.42)	114144
-30	-1 (200)	159929	289836	5288	42337	2241 (11.20)	20701
	-0.5 (117)	86135	143949	4833	30941	2057 (17.58)	15653
	-0.2 (93)	68730	111714	4351	24169	1853 (19.92)	13671

For varying values of the parameters `fs_open_cost` and `fs_extend_cost`, the values of the criteria for the `needlenuc` method .
